# The chronic care model: congruency and predictors among patients with cardiovascular diseases and chronic obstructive pulmonary disease in the Netherlands

**DOI:** 10.1186/1472-6963-12-242

**Published:** 2012-08-07

**Authors:** Jane Murray Cramm, Anna Petra Nieboer

**Affiliations:** 1 Institute of Health Policy & Management (iBMG), Erasmus University, Rotterdam, The Netherlands

**Keywords:** Disease management, Chronic care model, Chronic illness care, Quality, Primary care

## Abstract

**Background:**

The Chronic Care Model (CCM) achieved widespread acceptance and reflects the core elements of patient-centred care in chronic diseases such as CVD and COPD. Our aim is to assess the extent to which current care for CVD and COPD patients aligns with the CCM in Dutch healthcare practices in the early stages of implementing disease-management programmes, thereby revealing possible predictors that tell us whether certain patients are more likely to receive CCM-compliant care than others.

**Methods:**

We use a cross-sectional study, addressing CVD or COPD patients from 106 primary care practices in the Netherlands. Our response rate was 53%; i.e., 2,487 of 4,681 questionnaires were returned. The Patient Assessment of Chronic Illness Care (PACIC) was used to assess compliance with CCM. Quality of life was assessed with the Short-Form 36 Health Survey (SF-36) and we used the Hospital Anxiety and Depression Scale (HADS) to assess depressive symptoms. Multilevel regression models were performed to reveal predictors of high CCM compliance.

**Results:**

With a mean (SD) of 2.77 (0.86) in CVD patients and 2.89 (0.89) in COPD patients (*p* = 0.002), the PACIC sum score was lower than in previous studies conducted in HMOs in the US, but similar to a European primary care setting study. The PACIC score was associated with age and depressive symptoms in both patient groups.

**Conclusions:**

Younger and less depressed patients report higher PACIC scores, indicating that their care better aligns to the CCM.

## Background

Chronic diseases such as cardiovascular diseases (CVD) and chronic obstructive pulmonary disease (COPD) are major causes of death and disability worldwide with rising prevalence [1]. Despite advances in treatment, patients with chronic diseases do not always receive optimal care [2]. Current care for the chronically ill is often event-driven, despite solid evidence that a structured, proactive approach helps reduce the burden of many chronic diseases [3]. Their complexity necessitates a multi-faceted and integrated treatment tailored to patient needs [4], such as we have seen in disease management programmes (DMPs), which improve effectiveness and efficiency of chronic care delivery [5] by combining patient-related, professionally-directed, and organisational interventions [6,7]. In the Netherlands, DMPs are often based on the Chronic Care Model (CCM), which clusters six interrelated components of health care systems: health care organization, community linkages, self-management support, delivery system design, decision support, clinical information systems, and last, integration of the first five components. The idea is to transition chronic care from acute and reactive to proactive, planned, and population-based [5]. A recent literature review reaffirms the notion that successful improvement strategies in chronic disease care are consistent with the concept of the CCM [7]. The model provides a multidisciplinary approach to care for patients with chronic diseases, which involves the community and healthcare system and fosters communication between clinicians and well-informed patients. Unlike commercialized DMPs, it targets more than just patients [6].

No data are available to date showing the extent to which current primary care for the chronically ill is CCM-compliant but Glasgow *et al.* have developed the “Patient Assessment of Chronic Illness care” (PACIC) to assess it [8]. It is organised according to the key elements of the CCM and assesses the behaviour of professionals and practice teams from the patient’s perspective. The PACIC contains 20 items assessing five scale constructs: (i) “patient activation” assesses the extent to which the patient was motivated and supported by the physician to initiate changes; (ii) “decision support” assesses patient support via, for example, booklets and how satisfied they were with the organisation of their care; (iii) “tailoring” assesses the extent to which general instructions and suggestions were adapted to the patient’s personal situation; (iv) “problem solving” addresses how the physician dealt with problems which interfered with achieving predefined goals; and (v) “follow-up” addresses the frequency with which the process was followed up, if at all.

While Glasgow and colleagues could not reveal significant differences in the PACIC scores regarding gender, education and age [8,9], Rosemann and colleagues [10] found that younger, more highly educated, less depressed patients report higher PACIC scores, indicating that their care aligns to a higher degree with the CCM. Glasgow and colleagues [8,9] studied patients in hospital settings or HMOs; Rosemann and colleagues [10] investigated osteoarthritics in primary care settings.

We aim to increase our knowledge on the CCM in the primary care setting in the Netherlands and assess the congruency of care with the CCM in Dutch DMPs. And because of contradictions in relationships between PACIC scores and patient demographics, our second aim is to investigate whether care varies with socio-demographic or disease-related characteristics, revealing possible predictors of PACIC scores.

## Methods

### Study population

Our cross-sectional study included 2,487 patients (N = 4,681; response rate =53%) enrolled in thirteen newly-implemented DMPs in various regions in the Netherlands. Four DMPs are aimed at patients (n = 917) diagnosed with COPD and nine at diagnosed patients with CVD and patients at risk of CVD (n = 1,570) (hereafter called CVD patients). The DMPs comprise a variety of collaborations (mostly general practitioners, physiotherapists, and dieticians) undergoing internal practice redesign to improve chronic care management in 106 primary care practices. They address shortcomings in acute care models by identifying elements that encourage high-quality chronic disease care in the early stages of care for patients with COPD or CVD [11]. The professionals personally handed the questionnaire to patients at consultations or mailed it to patient’s homes. All non-respondents received a reminder and another copy of the questionnaire a few weeks later. The study was approved by the ethics committee of the Erasmus University Medical Centre of Rotterdam.

### Measures

Patients assessed chronic illness care (PACIC) with a 20-item questionnaire comprising five pre-defined subscales: patient activation (3 questions), delivery-system/practice design (3), goal setting/tailoring (5), problem solving/contextual (4), and follow-up/coordination (5). The five-point response scale ranged from ‘almost never’ to ‘almost always’ with higher scores indicating a more frequent presence of the respective aspect of chronic care. The PACIC score was the sum of participants’ responses divided by 20. Scores thus ranged from 1 to 5 with higher scores indicating a greater perception of involvement in self-management and receipt of chronic care counseling [8]. Cronbach’s alpha of patient activation = 0.83, delivery-system/practice design = 0.74, goal setting/tailoring = 0.79, problem solving/contextual = 0.86, follow-up/coordination = 0.75. Cronbach’s alpha of the overall PACIC scale was 0.93.

For scores of general health-related Quality of Life (QoL) we used the Short Form 36 Health Survey (SF-36) [12] to assess physical and mental health. Rules for item scoring and scales are available in the SF-36 Scoring Manual. All scales were transformed to values between 0 and 100 to allow scale and patient group comparability. Higher scores indicated a more positive rating. The Physical Component Score (PCS) and the Mental Component Score (MCS) were calculated. Selected items and weights derived from the general Dutch population [13] were then used to score the PCS and MCS.

We used the 7-item depression section of the Hospital Anxiety and Depression Scale (HADS) to assess symptoms of depression in CVD and COPD patients. All items were rated on a 4-point scale (0–3). Higher scores mean higher depressive symptomatology. The instrument has increasingly been used in inpatient, outpatient, primary care, and general population contexts [14-18]. The HADS has shown internal consistency, effective identification of depression, and concurrent validity for screening depression [14]. The Cronbach’s alpha of the HADS scale in our study was 0.83.

Education was assessed on six levels ranging from (1) no school or primary education (7 years of education or less) to (6) university degree (18 years of education or more).

### Analysis

Descriptive analysis included means and standard deviations. We tested for influence of primary care practices level on the PACIC score using a multilevel model. Results indicated that it significantly affected the scores (−2 log likelihood 4743.832 vs. 4541.423: *p* ≤ 0.001), thus calling for multilevel regression analyses. After calculating bivariate correlations, multilevel regression analyses were performed to reveal predictors of a high PACIC scale score. The hierarchical structure comprised patients (level 1) nested in primary care practices (level 2). All statistical analyses were conducted with SPSS version 17.0.

## Results

Table 1 displays characteristics of the study sample, which was nearly gender-equal. COPD patients were older than CVD patients (66.11 vs. 63.99), less likely to be married (74% vs. 67%), and less educated (1.92 vs. 2.46), scored lower on both mental and physical components of QoL, and had more symptoms of depression.

**Table 1:** **Sample characteristics of CVD patients (*n*** **= 1.570) and COPD patients (*n*** **= 917)**

	**Patient population**		p value*
	**CVD patients**	**COPD patients**	
	**% or Mean (SD)**	**% or Mean (SD)**	
Age	63.99 (10.19)	66.11 (10.57)	0.000
Gender (female)	48%	47%	0.523
Marital status (married)	74%	67%	0.000
Educational level (1–6)	2.46 (1.74)	1.92 (1.58)	0.000
*Quality of life*			
Physical Component Score	42.83 (10.47)	39.07 (10.16)	0.000
Mental Component Score	49.28 (9.75)	47.52 (10.95)	0.000
Depressive symptoms	4.27 (3.63)	5.23 (4.11)	0.000

*Notes.* *p-value = differences between groups established with *t* Test or Chi-square.

CVD, Cardio Vascular Diseases.

COPD, Chronic Obstructive Pulmonary Disease.

SD, Standard Deviation.

Table 2 shows the descriptive statistics of the individual scales of the PACIC scores for CVD and COPD patients. The average overall score of the PACIC was 2.77 (SD 0.86) for CVD patients and 2.89 (0.89) for COPD patients. COPD patients reported significantly higher PACIC scores on ‘delivery system design/decision support’ (*p* = 0.001), ‘goal setting/tailoring’ (*p* = 0.001), ‘problem solving/contextual’ (*p* < 0.001), and overall PACIC scores (*p* = 0.002).

**Table 2:** **Score distribution of the PACIC among CVD patients (*n*** **= 1.570) and COPD patients (*n*** **= 917)**

	**Patient population**				p Value*
	**CVD patients**		**COPD patients**		
	**Mean**	**SD**	**Mean**	**SD**	
Activation	2.91	1.15	3.00	1.18	0.085
Delivery system design/decision support	3.41	0.98	3.56	1.03	0.001
Goal setting/tailoring	2.61	0.93	2.74	0.97	0.001
Problem solving/contextual	2.66	1.10	2.91	1.15	≤ 0.001
Follow-up/coordination	2.24	0.96	2.22	0.96	0.689
Sum score PACIC	2.77	0.86	2.89	0.89	0.002

*Notes.* *t Test.

PACIC, Patient Assessment of Chronic Illness Care.

CVD, Cardio Vascular Diseases.

COPD, Chronic Obstructive Pulmonary Disease.

SD, Standard Deviation.

Correlations of the PACIC scores to patient characteristics, QoL, and incidence of depressive symptoms are displayed in Table 3. Among CVD patients significant correlations were found between age, depressive symptoms and the PACIC. PACIC scores of COPD patients significantly correlated with age, PCS, and depressive symptoms.

**Table 3:** **Correlations of patient variables with the PACIC among CVD patients (*n*** **= 1.570) and COPD patients (*n*** **= 917)**

	**Patient population**		
	**CVD patients**	**COPD patients**	
Age	−0.117***	−0.111**
Gender (female)	0.008	0.016
Marital status (married)	0.017	0.042
Educational level (1–6)	−0.052	0.059
*Quality of life*		
Physical Component Score	0.047	0.093**
Mental Component Score	−0.001	0.052
Depressive symptoms	−0.062*	−0.114***

*Notes.* *p < 0.05: **p < 0.01: ***p < 0.001 (two-tailed).

PACIC, Patient Assessment of Chronic Illness Care.

CVD, Cardio Vascular Diseases.

COPD, Chronic Obstructive Pulmonary Disease.

Table 4 displays the results of the multilevel regression analysis with the PACIC sum score as a dependent variable. Age was found as a predictor for CVD patients (*p* < 0.001), reflecting that younger patients had higher PACIC scores. More frequent depressive symptoms were associated with lower PACIC scores (*p* < 0.05). When we look at the results of the COPD population these revealed that having depressive symptoms was found to be a predictor for COPD patients (*p* < 0.001), reflecting that fewer depressive symptoms were associated with higher PACIC scores. Being younger was also associated with higher PACIC scores (*p* < 0.001). Although we found a significant relationship between the PCS and the PACIC score among COPD patients in the univariate analysis, the relationship was no longer significant in the multivariate analyses.

**Table 4:** **Predictors of the PACIC score assessed by multilevel regression analyses among CVD patients (*n*** **= 1.570) and COPD patients (*n*** **= 917)**

	**Patient population**			
	**CVD patients**		**COPD patients**	
	**B**	**SE**	**B**	**SE**
Age	−0.118***	0.003	−0.106***	0.030
Gender (female)	−0.036	0.052	0.007	0.029
Marital status (married)	−0.021	0.059	0.097	0.029
Educational level (1–6)	0.019	0.095	0.007	0.028
*Quality of life*				
Physical Component Score	−0.007	0.003	−0.023	0.032
Mental Component Score	−0.061	0.004	−0.070	0.044
Depressive symptoms	−0.082*	0.076	−0.119***	0.094
Explained variance				
Individual level	29%		36%	
Organisational level	14%		17%	

*Notes.* *p < 0.05: **p < 0.01: ***p < 0.001 (two-tailed). The Intra Class Correlation (ICC) value is 0.036

which means that 3,6% of individual-level variance can be explained by group membership.

PACIC, Patient Assessment of Chronic Illness Care.

CVD, Cardio Vascular Diseases.

COPD, Chronic Obstructive Pulmonary Disease.

SE, Standard Error.

## Discussion

The CCM has been promoted as a template of care for the chronically ill, aiming to substantially improve QoL [18,19]. Our study showed that certain patients rated aspects of their care that were consistent with the CCM more favourably. Being younger and less depressed increased the chance of a higher score on the PACIC for both COPD and CVD patient groups.

Evidence that interventions containing at least one CCM element could improve clinical outcomes as well as patient-relevant outcomes exists [20-22]. Adams *et al.* reported in a recent review that COPD patients who received interventions with two or more CCM components had lower rates of hospitalisations and emergency/unscheduled visits, and shorter hospital stays compared with control groups [23]. The studies, however, were conducted in hospital settings or HMOs and cannot easily be transferred to primary care settings. So far, only one study is available, showing that CCM elements can be implemented in small independent practices and result in improved care for diabetics [24]. The PACIC scores in our study were similar to a study in a German primary care setting for patients with osteoarthritis (men = 2.79, women = 2.67) [10]. The Dutch and German primary care setting PACIC scores were substantially lower than those of Glasgow *et al.* whose data was from an HMO setting [8,9].

In line with the findings of Rosemann and colleagues [10] PACIC scores were not correlated with disease severity in the multivariate analyses. MCS and PCS, reflecting different aspects of QoL of CVD and COPD patients, did not predict PACIC scores, suggesting that care delivered to chronically ill patients is not dominated by the severity of the chronic condition itself. Our results show COPD patients report care more congruent with the CCM compared to CVD patients. This may be explained by the stage of chronic care in the Dutch primary care setting: the COPD care standard (based on the CCM) was implemented in early 2010, while the recently-developed care standard for CVD patients has not yet been implemented in every health care practice. In addition, the CVD population included at-risk patients as well as patients with established disease. The at-risk patients may have had fewer interactions with their care teams and the teams may have put less effort into chronic care, which may also explain the lower average PACIC scores for the CVD population.

While Glasgow *et al.* could not reveal significant differences in the PACIC scores regarding patient characteristics in the HMO setting in the US [8,9], Rosemann *et al.* identified significant differences based on age, education, and depressive symptoms in the primary care setting in Europe [10]. We also found that younger and less depressed patients reported higher PACIC scores, indicating that their care better aligns with the CCM. Unlike Rosemann *et al.* we did not find a significant relationship between education and PACIC scores. This may be explained by disease duration. Patients in the Rosemann study had had osteoarthritis for about 14 years; most of our patients had been recently diagnosed. Different levels of education are most likely to result in differences in coping with a chronic condition over time. Educated people are expected to be better at self-management, getting necessary care, and compliance [10]. We thus expect to find significant relationships between education and PACIC score over time. The finding that younger, less depressed patients are more likely to report high PACIC scores could reflect differences in physician behaviour towards different patient groups and that such patients more actively seek CCM-compliant care, but the association is non-conclusive. The information is in any case valuable, since it suggests that ensuring that all patient groups benefit to the same extent from advances in chronic illness care is important in implementing CCM.

Our study is not without limitations. Most importantly, the data collected were cross-sectional and causal relationships could not be inferred. Depressive symptomatology may lead to a more negative appraisal of chronic care delivery, however, if patients receive high-quality chronic care this may also lead to less depressive symptoms among chronically-ill patients. Longitudinal data is necessary to disentangle the dynamic relationship between depressive symptomatology and high-quality chronic care delivery. We also expect to find a dynamic relationship between QoL and chronic care delivery. Since we included patients recently enrolled in newly-implemented DMPs we investigated the influence of QoL on patient’s assessment of chronic illness care delivery. There is, however, also evidence that higher levels of chronic care delivery results in improved QoL [18-22]. Again, longitudinal data is necessary to disentangle the dynamic relationship between QoL and chronic care delivery. Finally, our sample of CVD and COPD patients limits generalizing study findings to other diseases. Our findings do, however, confirm those of Rosemann *et al.* among patients with osteoarthritis [10]. The strength of our study is its reasonably large and representative sample of primary care practices.

Based upon the work of Glasgow and colleagues [8] there is adequate evidence to support the use of the survey to measure the CCM. However, further development and refinement of its psychometric properties is needed and some studies point to possible limitations of the PACIC instrument. We used the PACIC as a reflective measure to assess patients’ assessment of chronic care delivery. In accordance with the findings of Glasgow and colleagues [8] who developed the instrument, we assumed the PACIC to reflect the underlying construct of chronic care delivery. Spicer and colleagues [25], however, argue that the PACIC is a formative measure and scores on the items cause or form the respondent’s status with respect to the construct. Following their reasoning this may indicate that the level of chronic care delivery also emerges or is formed as a result of patients’ responses. Gugiu and colleagues [26] argued that the PACIC is actually unidimensional instead of the subscale construct and scoring of the PACIC should be changed to an 11-point scale ranging from 0% to 100% by units of 10% instead of using a 5-point response scale. More research is necessary focusing on the instrument’s validity and reliability.

## Conclusion

The PACIC can be used as a monitoring tool to assess the quality of chronic care delivery. These study findings show that it is important to realize that the assessment of chronic care delivery by patients may not only depend on the care patients received, but may also depend on patient characteristics such as age and depressive symptoms. The finding that younger and less depressed COPD and CVD patients are more likely to receive CCM-compliant care suggests that a challenge to implementing CCM in primary care is to ensure that all patients benefit equally. A question we might ask is: Are approaches that are appropriate to younger and less depressed patients different from those used for older or more depressed patients?

## Competing interests

The authors declare that they have no competing interests.

## Author’s contribution

AN drafted the design for data gathering. JC and AN were involved in acquisition of subjects and data, performed statistical analysis and interpretation of data. JC drafted the manuscript and AN helped drafting the manuscript and contributed to refinement. Both authors have read and approved its final version.

## Pre-publication history

The pre-publication history for this paper can be accessed here:

http://www.biomedcentral.com/1472-6963/12/242/prepub
